# Optimal reference genes for normalization of qPCR gene expression data from proton and photon irradiated dermal fibroblasts

**DOI:** 10.1038/s41598-018-30946-0

**Published:** 2018-08-23

**Authors:** Steffen Nielsen, Niels Bassler, Leszek Grzanka, Jan Swakon, Pawel Olko, Christian Nicolaj Andreassen, Jan Alsner, Brita Singers Sørensen

**Affiliations:** 1 Department of Experimental Clinical Oncology, Aarhus University Hospital, Aarhus, Denmark; 2 Medical Radiation Physics, Department of Physics, Stockholm University, Stockholm, Sweden; 3 Proton Radiotherapy Group, Institute of Nuclear Physics Polish Academy of Sciences, Krakow, Poland

## Abstract

The transcriptional response of cells exposed to proton radiation is not equivalent to the response induced by traditional photon beams. Changes in cellular signalling is most commonly studied using the method Quantitative polymerase chain reaction (qPCR). Stable reference genes must be used to accurately quantify target transcript expression. The study aim was to identify suitable reference genes for normalisation of gene expression levels in normal dermal fibroblasts irradiated with either proton or photon beams. The online tool RefFinder was used to analyse and identify the most stably expressed genes from a panel of 22 gene candidates. To assess the reliability of the identified reference genes, a selection of the most and least stable reference genes was used to normalise target transcripts of interest. Fold change levels varied considerably depending on the used reference gene. The top ranked genes *IPO8*, *PUM1*, *MRPL19* and *PSMC4* produced highly similar target gene expression, while expression using the worst ranked genes, *TFRC* and *HPRT1*, was clearly modified due to reference gene instability.

## Introduction

Proton beam therapy induces an altered stress response in both cancerous and normal cells compared with traditional photon beam therapy^1^. Previous research indicates that molecular mechanisms such as DNA repair and epigenetic regulation may be affected differently by various radiation qualities^2–6^. Furthermore, the transcriptional response may also be influenced by position in the proton beam as the linear energy transfer (LET) increases along the proton track^7^. Therefore, it seems unlikely that the differences in transcriptional response to photon and proton irradiation can be equated by modulating the radiation dose. Potential changes in cellular signalling may lead to alternate outcomes regarding both tumour progression and normal tissue complication development thereby stressing the importance of investigating proton radiobiology.

Currently, quantitative real-time polymerase chain reaction (qPCR) is the most widely used technique for studying differential expression of specific genes following an experimental treatment exposure. Implementation of the qPCR technique has greatly contributed to improve knowledge of cellular responses to various treatment modalities using numerous tumour and normal tissue models^8–11^. The comparative C_T_ method is the most widely used strategy for qPCR data analysis^12^. The method relies on endogenous reference genes to determine relative expression of target transcripts. Expression of ideal reference genes is unaffected by the treatment exposure and stable across the investigated cell types. Genes such as *GAPDH* and *ACTB* have traditionally been used as reference genes without much consideration to their stability under the specific conditions of a given experiment^13^. However, the stability of *GAPDH* and *ACTB* have been shown to be affected by a number of experimental exposures and it is unlikely that any universal reference gene exists^14^. Optimal reference genes for qPCR data analysis have been suggested for multiple tumour cell lines irradiated with particle and photon beams but, to our knowledge, there have not been any reported reference genes for normal cells^15^.

The present study aimed to identify and evaluate stably expressed reference genes in proton and photon irradiated primary dermal fibroblast cultures. The genes *IPO8*, *MRPL19*, *PSMC4*, and *PUM1* performed best in the applied selection algorithms and produced highly similar relative quantification of target transcripts.

## Results

In the present study, new qPCR gene expression data for candidate reference genes and target transcripts of interest are presented. The data are derived from material generated in a previous study, where qPCR data for other transcripts were reported. LET values presented here were reported in the previous study^7^.

### Depth-dose profile and LET values

**Figure 1:**
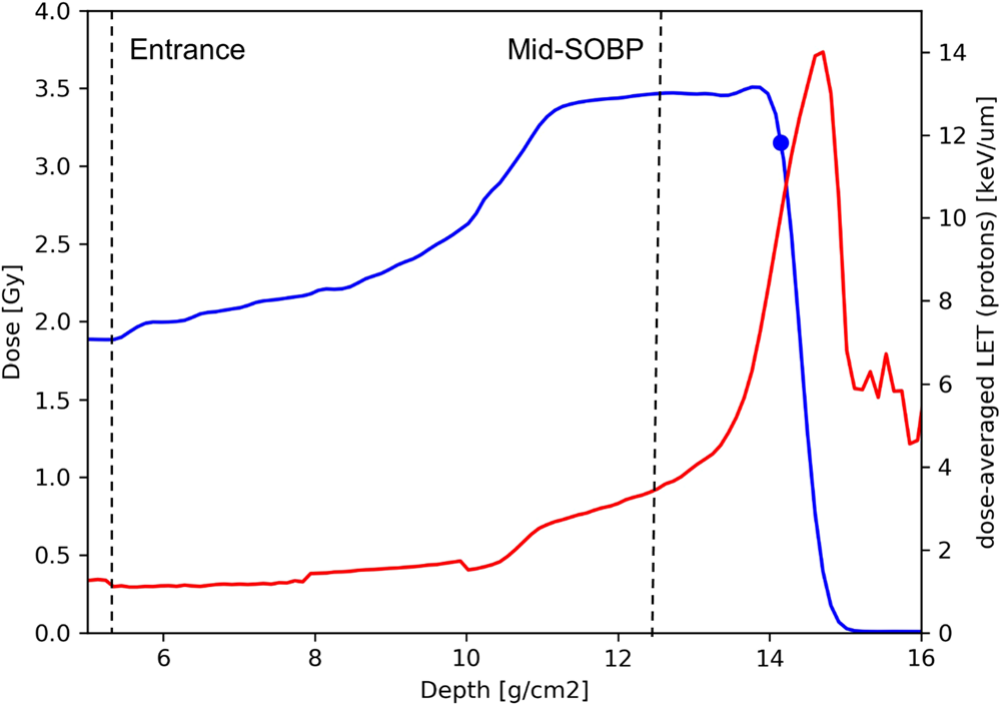
Depth-dose profile of the proton beam used to irradiate fibroblasts in the SOBP distal edge. The blue line shows the dose deposition and the red shows how the dose-averaged LET increases along the proton track. The blue dot marks the position of the culture flasks. The dotted lines approximately mark the position depth of flasks in the entrance and mid-SOBP groups. Equivalent profiles for the entrance and mid-SOBP groups were scaled to deliver 3.5 Gy(RBE) in their respective positions.

Primary fibroblasts were positioned in the entrance, the middle of the SOBP and the distal edge of the SOBP using three different depth-dose plans to deliver 3.5 Gy(RBE) × 3 fractions in all groups but with increasing LET along the depth-dose profile. Dose-averaged LET values were 1.1, 3.3 and 8.4 keV/µm for protons only, ignoring secondary particles, in the entrance, mid-SOBP and SOBP distal edge groups. The depth-dose profile for cells irradiated with the highest LET proton beam is displayed in Fig. 1.

### Overview of C_T_ values for candidate reference genes

Nine primary dermal fibroblast cultures were used in the treatment and control groups. Descriptive statistics are provided for the C_T_ values of all the included candidate reference genes in Table 1. *PMM1* and *RPL37A* had the smallest coefficient of variation at 1.7%, while *ACTB* and *GAPDH* had the highest degree of dispersion with CVs at 3.1% and 3.2%. *RPL37A* had the smallest C_T_ range with 1.9 cycles from the lowest to the highest C_T_ value across all 45 samples. Mean C_T_ values for all candidates ranged from 17.1 to 27.3, thus covering most of the range for potential target transcripts.

**Table 1:** Basic descriptive statistics for the 22 candidate genes with associated identification number from Applied Biosystems part of ThermoFisher Scientific.

Gene	C_T_ range	C_T_ min	C_T_ max	C_T_ mean	CV %	Identification
*PMM1*	1.9	24.5	26.3	25.1	1.7	Hs00160195_m1
*RPL37A*	1.4	18.1	19.5	18.8	1.7	F: TGTGGTTCCTGCATGAAGACA R: GTGACAGCGGAAGTGGTATTGC P: TGGCTGGCGGTGCCTGGA
*NDFIP1*	1.8	22.1	23.9	23.2	1.9	Hs00228968_m1
*CHCHD1*	2.3	23.7	26.0	24.9	1.9	Hs00415054_m1
*POLR2A*	1.8	22.2	23.9	23.1	1.9	Hs01108291_m1
*PUM1*	2.1	23.6	25.7	24.9	2.0	Hs00472881_m1
*GUSB*	2.2	23.8	26.0	24.9	2.0	Hs99999908_m1
*SF3A1*	2.4	23.1	25.5	24.5	2.0	Hs01066327_m1
*MRPL19*	2.4	23.3	25.7	24.9	2.1	Hs00608519_m1
*PSMC4*	2.4	23.6	25.9	24.9	2.1	Hs00197826_m1
*TBP*	2.6	24.1	26.7	25.8	2.1	Hs00427621_m1
*IPO8*	2.6	23.8	26.3	25.4	2.2	Hs00183533_m1
*B2M*	1.7	17.7	19.4	18.6	2.3	Hs00984230_m1
*CALM2*	2.0	19.3	21.3	20.5	2.3	F: GAGCGAGCTGAGTGGTTGTG R: AGTCAGTTGGTCAGCCATGCT P: TCGCGTCTCGGAAACCGGAGC
*HMBS*	2.8	25.4	28.2	27.3	2.3	Hs00609293_g1
*RPLPO*	2.6	18.1	20.7	19.2	2.4	Hs00420895_gH
*ACTR3*	2.3	20.7	23.0	22.0	2.4	Hs01029161_m1
*TFRC*	2.5	24.0	26.5	25.5	2.6	Hs00951083_m1
*PPIA*	2.3	17.9	20.3	19.3	2.9	Hs99999904_m1
*HPRT1*	3.1	22.6	25.7	24.7	3.1	Hs99999909_m1
*ACTB*	2.3	15.9	18.2	17.1	3.1	Hs01060665_g1
*GAPDH*	2.0	16.2	18.3	17.2	3.2	Hs02758991_g1

Genes are ranked by CV%. Summary statistics are for a total of 45 samples, 9 cell lines used in 5 groups including the control group. The oligo sequences are not made public by Applied Biosystems. Primer and probe sequences are listed for the assays from DNA Technology.

### Identification of the most stable reference genes

**Figure 2:**
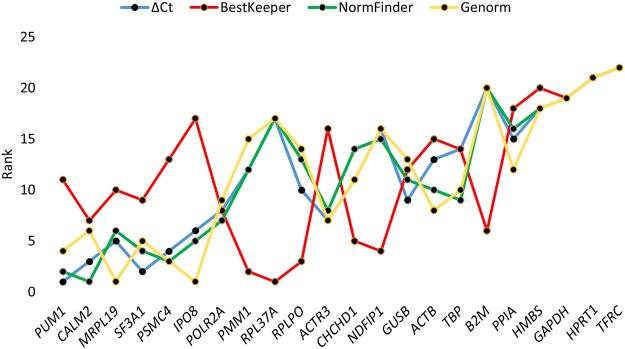
Ranking of the 22 reference gene candidates. The most stable genes, as identified by the RefFinder overall scoring, are shown left to right on the x-axis. The curves show the achieved rank in the four algorithms ΔCt method, Genorm, NormFinder and Bestkeeper. Nine primary dermal fibroblast cultures were used in each of the four treatment groups and in the untreated control group.

The online tool RefFinder was used to generate an overall ranking of the most stable reference genes based on the scores from the Genorm, NormFinder, BestKeeper and ΔCt algorithms (Fig. 2). The genes *PUM1*, *CALM2*, and *MRPL19* had the best overall scores. The algorithms Genorm, NormFinder and ΔCt produced similar results, while BestKeeper identified other genes as ideal for normalization. NormFinder found the combination of *PUM1* and *CALM2* optimal for normalization, these were identified as best and third best by the ΔCt method and Genorm ranked them fourth and sixth. Genorm identified *IPO8, MRPL19* and *PSMC4* as the most stably expressed genes across all samples. NormFinder ranked them third, fifth and sixth most stable, while the ΔCt method ranked them fourth, fifth, and sixth. BestKeeper identified *RPL37A* and *PMM1* as optimal reference genes. All of the algorithms identified *TFRC* as the least stable reference gene with *HPRT1* as the second least stable. The most frequently used reference gene *GAPDH* was ranked third least stable of the 22 included candidates.

### Assessing the impact of reference gene selection

**Figure 3:**
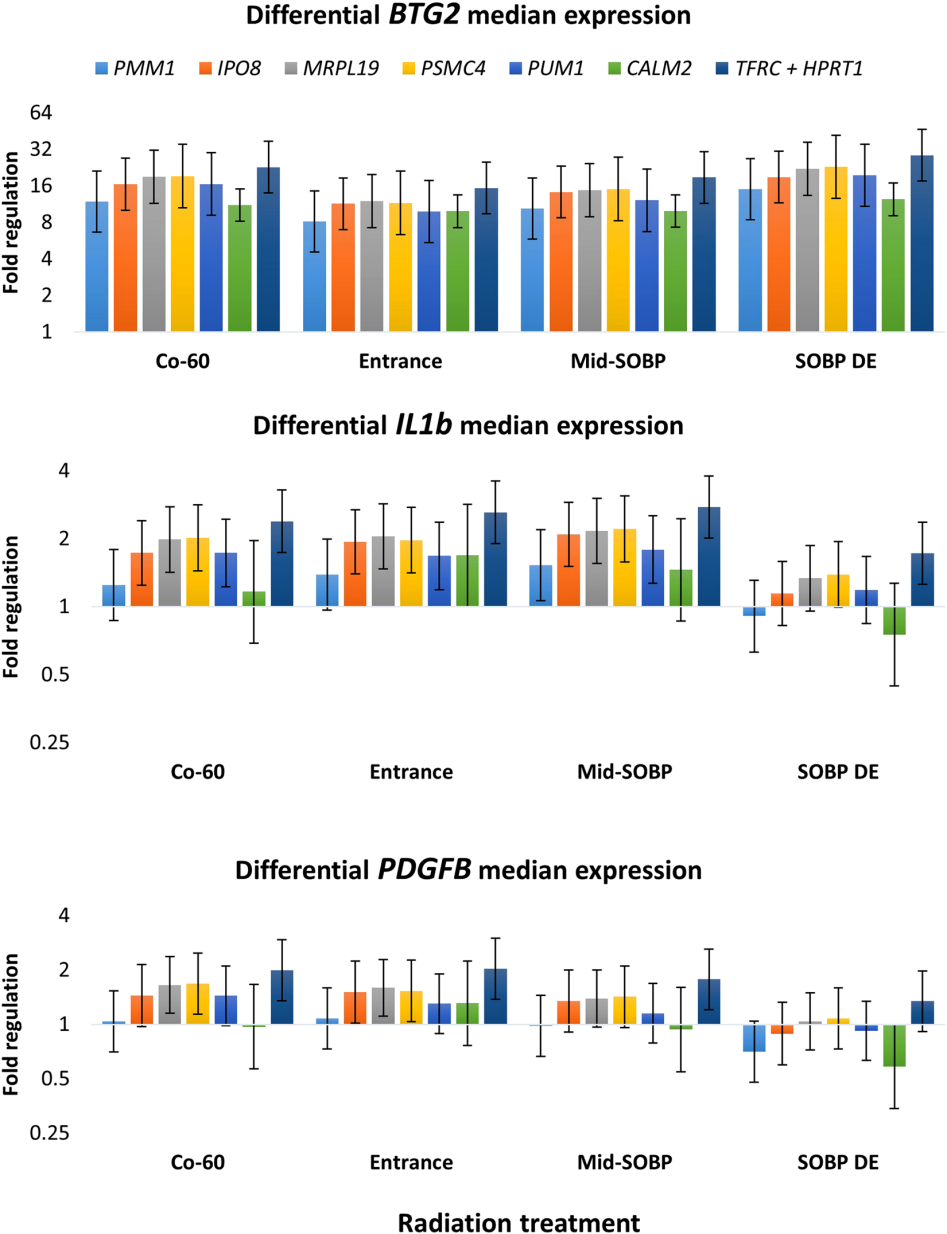
Estimated median fold change levels of the genes *BTG2*, *IL1b* and *PDGFB*. Median fold changes are shown for the four radiation treated groups relative to controls with 95% confidence intervals. The same 12 cell lines were used in each group (n = 12). Six of the best performing reference genes and the average of the two worst performing reference genes were used to calculate median fold change to assess the impact of using different reference genes.

It is an inherent problem in identification of optimal reference genes that selected reference genes cannot be clearly validated. Instead, reliability can be evaluated by determining how fold changes of target transcripts are influenced by different reference genes. Here, *BTG2, IL1b* and *PDGFB* were chosen as target transcripts as they are involved in critical molecular mechanisms such as regulation of cell proliferation, inflammatory response, angiogenesis, and differentiation. Fold changes were calculated for target transcripts by normalisation with the reference genes *IPO8*, *PMM1*, *MRPL19*, *PSMC4, PUM1* and *CALM2* and by using the average of *TFRC* and *HPRT1*. *IPO8*, *PMM1*, *MRPL19*, *PSMC4, PUM1* and *CALM2* were chosen as they were recommended by the different algorithms in RefFinder, thus not selected by the overall ranking. The average of *TFRC* and *HPRT1* is also included in the evaluation as the genes performed worst in all the algorithms and thereby provides a basis for comparison. Using *IPO8*, *MRPL19*, *PSMC4* or *PUM1* as reference genes produced similar fold changes for all target genes within respective treatment groups. *PMM1* and *CALM2* generated smaller fold changes than when *TFRC* and *HPRT1* were applied (Fig. 3).

It is also of great interest to investigate how differences between treatment groups are affected by normalisation with different reference genes. The influence of radiation quality on target transcript regulation is determined by performing a one-way Analysis of Variance (ANOVA) with a random patient effect to test for no difference in estimated median fold change between treatment groups for *BTG2*, *IL1b*, and *PDGFB*. The differences in fold changes between groups were quantified by post-hoc, pairwise comparisons. *CALM2* and *PUM1* produced the smallest and biggest differences between treatment groups for all three target transcripts. For instance, *BTG2* fold change was more strongly induced in the SOBP distal edge group than in the entrance group no matter what reference gene was used. Expression was increased by 26% (95%-CI: 9–45%, p < 0.0001) when *CALM2* was used as reference and 99% (14–248%, p = 0.016) when *PUM1* was used as reference.

## Discussion

Three of the four selection algorithms employed in the RefFinder tool generally ranked *IPO8*, *MRPL19*, *PSMC4*, *PUM1,* and *CALM2* as the most stable. However, BestKeeper identified *PMM1* and *RPL37A* as the most stable genes. Those two genes also had narrow C_T_ ranges across all samples and the lowest Coefficients of Variation. Rydbirk *et al*. also reported that BestKeeper produced a ranking that deviated from the other algorithms^16^. A major drawback of using these selection algorithms is that they cannot determine whether the most stable genes are actually sufficiently stable for accurate analysis. To limit the effects of using an inappropriate reference gene, the average C_T_ of 3 or more reference genes should generally be employed as a normalization factor^17^.

It is evident in the present study that the choice of reference gene can affect fold change levels of target transcripts to such an extent that it will influence the biological interpretation. This is observed when comparing fold change levels of *IL1b* when normalised with either the average of *TFRC* and *HPRT1* or *CALM2*. *TFRC* and *HPRT1* normalized fold change levels always show a substantial upregulation of *IL1b*, while *CALM2* normalized expression is either unaffected, up- or downregulated, thus resulting in two different conclusions on how *IL1b* is regulated in fibroblasts following radiation exposure. Similar observations can be made for *PDGFB*. While *BTG2* is conclusively upregulated by irradiation no matter what reference gene is used, the extent of upregulation is strongly affected by choice of reference gene.

Interestingly, the average C_T_ of *PUM1* and *CALM2* were identified by NormFinder as the optimal normalization factor although they produced highly varying fold change levels in the present study. Actually, normalization with either *PUM1* or *CALM2* resulted in the biggest and smallest increase of *BTG2* median fold change between the entrance and SOBP distal edge treatment groups with 99% and 26%, respectively. Critically, when using any of the two genes for normalization, a significant increase in *BTG2* expression could be identified in the SOBP distal edge group compared with the entrance group, thus not drastically altering the conclusion on how different LET proton beams influence regulation of *BTG2*.

Sharungbam *et al*. have previously reported *RPLP0*, *UBC*, *PPIA*, *TBP* and *PSMC4* to be the most stable reference genes from a panel of 32 candidates^15^. These were identified using three tumour cell lines treated with photon, proton and carbon-ion irradiation. Except for *UBC*, the other genes were included in the candidate panel in the present study and only *PSMC4* was ranked among the best performing reference genes. This discrepancy may have multiple explanations. The potentially substantial cytogenetic differences between established tumour cell lines and primary normal cell lines could alone lead to identification of different optimal reference gene sets. Furthermore, carbon-ion irradiation used in the study by Sharungbam *et al*. appears to have a unique impact on molecular processes which will affect gene expression thereby leading to identification of reference genes suitable for photon, proton, and carbon-ion irradiation^18^. Finally, the differences in composition of the candidate gene panels could affect the outcome. The Genorm algorithm, also used by Sharungbam *et al*., employs stepwise exclusion of the least stable candidate gene based on the stability score *M*. For a specific candidate gene, *M* is the average pairwise variation with all other candidate genes^17^. The composition of the candidate gene panel can especially impact selection of optimal reference genes if any panel genes are co-expressed^19^.

In the present study, it was demonstrated that identifying appropriate reference genes for comparative C_T_ analysis is critical in order to obtain robust results. Choice of reference genes may affect the quantification of target transcript fold change levels. *IPO8*, *MRPL19*, *PSMC4* and *PUM1* were among the most stably expressed genes in dermal fibroblasts exposed to different radiation qualities as identified by the most commonly used selection algorithms. The identified genes comprise a suitable normalization factor in the comparative C_T_ method and the genes are suggested as candidates for future proton radiobiology research where qPCR is employed to determine differential gene expression in non-malignant cells.

## Materials and Methods

### Primary dermal fibroblast cultures

The primary dermal fibroblast cultures used in the present study were available from a biobank previously generated at the Aarhus University Hospital^20^. Fibroblast cultures were established using skin biopsies taken from the forearm of patients about to undergo treatment for head and neck cancer. At the time, patients provided informed consent for their samples to be used in research. The use of the fibroblast biobank for this study was approved by the Danish National Committee of Medical Ethics (case no. 1-10-72-170-15) and the study is performed in accordance with their guidelines.

### Cell culturing

AmnioMAX-C100 Basal Medium supplemented with 7.5% AmnioMAX-C100 Supplement, 7.5% HyCloneTM fetal bovine serum, 1% penicillin–streptomycin and 1% L-Glutamine 200 mM (ThermoFisher Scientific, Waltham, MA, USA) was used for all cell culturing. Incubation was performed at 37 °C with 5% CO_2_ and around 90% relative humidity. Cultures were passaged twice before approximately 200,000 cells were seeded in T25 flasks and cultured for 3 days. Thereafter, growth medium was changed and cultures were incubated for another 24 hours. The culture flasks had to be irradiated in a vertical position hence the flasks were filled with medium immediately prior to delivery of the first of three fractions. The flasks were kept vertical and filled with medium until the cells were harvested 2 days later. Surface adherence, growth rate and lethality of the fibroblasts were previously verified to be identical in the vertical position compared with the same fibroblasts kept under standard culturing conditions.

### Proton scanning beam and Cobalt-60 photon irradiation

Irradiation was performed at the Institute of Nuclear Physics – Polish Academy of Sciences, Krakow, Poland. To deliver the radiation dose, three culture flasks per fraction were placed vertically in a water-filled polymethylmethacrylate (PMMA) phantom. All cell cultures were irradiated following the same radiation regimen of 3.5 Gy(RBE) × 3 fractions with 24-hour intervals and using a RBE of 1.1 to scale proton doses. The IBA ProteusPLUS gantry with a Pencil Beam Scanning (PBS) dedicated nozzle was used for all proton irradiations. Fibroblast cultures were placed in 3 different positions of the proton depth-dose profile. They were placed in the entrance, SOBP centre or at the SOBP distal edge of different depth-dose profiles in order to deliver the same dose in each group. X-ray imaging and a Markus Ion Chamber were used to position the phantom and verify the depth-dose profiles. Total uncertainty in dose delivery was lower than 2.5%. Cobalt-60 beam irradiation was delivered using the Theratron 780E system with a 0.307 Gy/min dose rate. Additional set-up details are previously reported elsewhere^7^.

### RNA purification and qPCR

Cell lysis and RNA purification were performed with the miRNeasy mini kit (Qiagen, Hilden, Germany). Cells were lysed with the Quiazol Lysis Reagent 2 hours after the last fraction was delivered. Residual genomic DNA was enzymatically digested in the purification process. Qubit^TM^ 3.0 Fluorometer with Qubit RNA Broad-Range Assay Kit was used to determine the RNA concentration in samples. The High-Capacity cDNA Reverse Transcription Kit (ThermoFisher Scientific) was used to generate cDNA from approximately 2 µg of RNA according to the manufacturer’s instructions. Transcripts were quantified using Taqman Gene Expression Assays (ThermoFisher Scientific) except for the *CALM2* and *RPL37A* transcripts, where probes and primers from DNA technology were used (DNA Technology, Risskov, Denmark). Taqman assays spanning exon-exon junctions were selected when available to minimise risk of genomic DNA contamination. The 7900HT Fast Real-Time PCR System was used to perform qPCR (ThermoFisher Scientific).

### Identifying optimal reference genes and statistical analysis

Twenty-two potentially suitable reference genes commonly used for qPCR data normalization were chosen as candidates (Table 1). The online tool RefFinder (currently available at: http://150.216.56.64/referencegene.php) was used to evaluate the expression stability of reference gene candidates by applying the four algorithms: Genorm^17^, NormFinder^19^, BestKeeper^21^ and the ΔCt method^22^. RefFinder also produced an overall performance ranking of the candidate genes based on the scores obtained in the four algorithms. The signalling pathways and functions of the included candidate genes were examined to limit the risk of co-dependent expression, which potentially could skew the selection of optimal reference genes in some of the applied algorithms.

Regulation of target genes in response to irradiation is described here as fold changes. Fold changes of the three target genes *BTG2 (*Hs00198887_m1), *IL1b (*Hs01555410_m1), and *PDGFB (*Hs00966522_m1) were produced using selected reference genes to assess how they perform in normalisation. The comparative C_T_ method was used to calculate fold changes^12^:

$${2}^{-{\rm{\Delta }}{\rm{\Delta }}\mathrm{Ct}}\,{\rm{where}}\,{{\rm{\Delta }}{\rm{\Delta }}{\rm{C}}}_{{\rm{T}}}={{\rm{\Delta }}{\rm{C}}}_{{\rm{T}}{\rm{Irradiation}}}-{{\rm{\Delta }}{\rm{C}}}_{{\rm{T}}{\rm{Normal}}{\rm{Control}}}$$

Log-transformation was performed on fold changes to approach a normal distribution. Analysis of Variance (ANOVA) with a random patient effect was performed to test for no difference between treatment groups. Pairwise comparisons were used to identify differences in median fold changes between groups. Stata software, version 14.2, StataCorp (College Station, Texas, USA) was used for data analysis.

### Monte-Carlo simulations

Particle transport simulations were performed to obtain detailed spatial distribution of dose and LET. For this purpose, the Monte-Carlo particle transport code SHIELD-HIT12A code version 0.7.2. was used^23^. To obtain satisfactory statistical convergence of the results this code was run in parallel on 200 CPU cores on Prometheus supercomputer, on each core, trajectories of 10^6^ primary protons and all secondary particles were transported. Simulated geometry consisted of a simplified model of flasks filled with medium, cells and water phantom. The initial scanning proton beam spot weights, positions, and initial kinetic energies were obtained from the accelerator control file. Output parameters were scored on a rectangular mesh, with 1 × 1 cm² cross-section and 1 mm resolution in the beam axis direction.

## Data Availability

The datasets generated and analysed during the current study are available from the corresponding author on reasonable request.
